# Late Neurological and Cognitive Sequelae and Long-Term Monitoring of Classical Hodgkin Lymphoma and Diffuse Large B-Cell Lymphoma Survivors: A Systematic Review by the Fondazione Italiana Linfomi

**DOI:** 10.3390/cancers13143401

**Published:** 2021-07-07

**Authors:** Silvia Franceschetti, Maria Antonietta Annunziata, Giulia Agostinelli, Chiara Gerardi, Eleonora Allocati, Carla Minoia, Attilio Guarini

**Affiliations:** 1 Haematology Unit, Ospedale Civile di Legnano, ASST Ovest Milanese, 20025 Legnano, Italy; 2 Oncological Psychology Unit, Centro di Riferimento Oncologico di Aviano (CRO) IRCCS, 33081 Aviano, Italy; annunziata@cro.it (M.A.A.); giulia.agostinelli@cro.it (G.A.); 3 Istituto di Ricerche Farmacologiche “Mario Negri” IRCCS, 20156 Milano, Italy; chiara.gerardi@marionegri.it (C.G.); eleonora.allocati@marionegri.it (E.A.); 4 Hematology Unit, IRCCS Istituto Tumori “Giovanni Paolo II”, 70124 Bari, Italy; carlaminoia@libero.it (C.M.); attilioguarini@oncologico.bari.it (A.G.)

**Keywords:** survivors, classical Hodgkin lymphoma, diffuse large B-cell lymphoma, neuropathy, cognitive impairment, fatigue, anxiety, depression, quality of life, systematic review

## Abstract

**Simple Summary:**

The last 25 years have seen a significant increase in the number of lymphoma survivors. This review was carried out to examine the data currently available on the incidence of some of the consequences most reported by this population: peripheral neuropathy, cognitive impairment, fatigue, and anxiety and depression. This review also investigated any follow-up strategies or monitoring implemented. The 35 articles included in the final analysis provided an idea of what the incidence of these sequelae may be in long-term survivors of classical Hodgkin lymphoma and diffuse large B-cell lymphoma. Despite methodological limitations encountered in the literature search, the Authors attempted to summarize the available evidence and provide support to clinical practice. This systematic review represents the basis for designing future studies with a longitudinal trial design and examining more homogeneous populations to assess and monitor these dimensions over time in clinical practice and to respond promptly to the needs of lymphoma survivors.

**Abstract:**

Background: The continuously improving treatment outcome for classical Hodgkin lymphoma (cHL) and diffuse large B-cell lymphoma (DLBCL) over the last 25 years has led to a high number of long-term survivors. The impact of treatment, however, can sometimes be dramatic and long-lasting. Focusing on peripheral neuropathy (PN), cognitive impairment, fatigue, anxiety, and depression, researchers of the Fondazione Italiana Linfomi conducted a systematic review of the literature to collect the available data on sequelae incidence as well as evidence of follow-up strategies for long-term cHL and DLBCL survivors. Methods: The review was carried out under the methodological supervision of the Istituto di Ricerche Farmacologiche “Mario Negri”, Milan, Italy. The literature search was conducted on three databases (MEDLINE, Embase, and the Cochrane Library) updated to November 2019. The selection process and data extraction were conducted according to the Preferred Reporting Items for Systematic Reviews and Meta-Analyses (PRISMA) guidelines. Results: A total of 2236 abstracts were screened, 247 full texts were analyzed, and 35 papers were included in the final analysis. Fatigue was the most extensively studied among neuropsychological sequelae, with a mean prevalence among cHL survivors of 10–43%. Although many of the papers showed an increased incidence of PN, cognitive impairment, and anxiety and depression in long-term cHL and DLBCL survivors, no definite conclusions can be drawn because of the methodological limitations of the analyzed studies. No data on monitoring and follow-up strategies of PN and other neuropsychological sequelae were highlighted. Conclusions: Based on our findings, future studies in this setting should include well-defined study populations and have a longitudinal trial design to assess the outcomes of interest over time, thus as to structure follow-up programs that can be translated into daily practice.

## 1. Introduction

Classical Hodgkin lymphoma (cHL) and diffuse large B-cell lymphoma (DLBCL) are now considered curable diseases in more and more cases. Indeed, thanks to successful treatment, the last 25 years have seen an increasing number of long-term survivors [1]. However, anti-cancer treatments can sometimes lead to several consequences that have a severe, long-lasting impact on quality of life, comorbidities, and overall survival [2].

Among the toxicities reported after treatment for lymphoma, neuropsychological sequelae can be disabling and irreversible and may occur during treatment and/ or follow-up. These present a range of clinical manifestations, including cancer-related fatigue, neurotoxicity, and mood disorders.

cHL often develops in young adults, who have a long life expectancy and in whom persistent fatigue, mood disturbances, and impaired cognition may have a dramatic impact on quality of life, affecting their working and learning capacities and multiple aspects of their social life.

Regarding neurotoxicity, neuropathy or peripheral neuropathy (PN) is the best characterized; it is defined as the condition arising from the damage and the dysfunction of the peripheral nerves—the motor, sensory, and autonomic nerves that connect the brain and the spinal cord to the rest of the body. The severity of neuropathy ranges from discomfort to being severely debilitating. Patients often feel abnormal sensations, such as tingling, pain, or numbness, and may complain about difficulty in walking, dropping things, or feeling like they are wearing gloves or stockings when in fact, they are not. Internal organs can be affected as well, and patients may experience diarrhea or constipation or cardiovascular effects. Both old and new lymphoma treatment strategies, mainly vinca alkaloids or brentuximab-vedotin (BV), can often be complicated by PN [3,4], forcing clinicians to promptly recognize and manage this complication. However, little is known about the persistence of PN symptoms among lymphoma survivors or about its impact on health-related quality of life (HRQoL).

HRQoL is a multidimensional construct, which reflects the World Health Organization’s definition of health as “a state of complete physical, mental and social well-being” [5,6]. There are several tools that assess the multidimensional construct or focus on single domains or symptoms such as fatigue.

Cancer-related fatigue is a lasting subjective feeling of physical, emotional, or intellectual exhaustion that cannot be explained by previous activities. This severe exhaustion compromises the everyday life of affected cancer patients and survivors. While persistent fatigue is frequently reported by cHL patients during both treatment and follow-up, it can remain a considerable problem even years after successful lymphoma treatment [6].

A more recently defined concept is cancer-related cognitive impairment (CRCI), an adverse effect of various malignancies and their treatments commonly reported by adult survivors [7]. CRCI is usually characterized by a decrease in memory, attention, executive function, and processing speed [8]. Many assessment tools, divided into two general types, are used to determine CRCI incidence: subjective self-assessment questionnaires and objective evaluation tests performed by a neuropsychologist. Most CRCI studies have been performed on breast cancer patients; little is known about CRCI in adult long-term cHL and DLBCL survivors.

Mood disturbances are also common in cancer survivors, with anxiety and depression being the most frequently reported. Studies have shown that undetected anxiety and depression may significantly affect the quality of life and even increase the mortality of cancer patients [9,10].

Although major lymphoma guidelines have included general recommendations for neurotoxicity management and psychological sequelae, in particular for cHL survivors, well-defined strategies for prevention, early diagnosis, and treatment are still lacking [11].

The National Comprehensive Cancer Network (NCCN) Guidelines for Survivorship, which address all long-term cancer patients [11], suggest a brief inventory questionnaire to assess the domains of cognitive function, mood disturbances, and fatigue. However, no effective brief screening tool for CRCI has yet been developed, and there is limited evidence to guide the management of this condition. Nevertheless, as they do for fatigue management, the Guidelines suggest searching for all the potentially reversible factors that may contribute to persistent CRCI and also give some advice on lifestyle interventions.

Studies on long-term complications of lymphoma treatment often include pediatric and young adult patients or patients who are not disease-free or with less than 5 years of follow-up after treatment. We tried to limit our study population by including only adults cured after cHL or DLBCL treatment, defined as patients who were diagnosed and treated at adult age (>18 years old) and have been treatment-free for at least 5 years.

Given this, Fondazione Italiana Linfomi (FIL) researchers conducted a systematic review of the literature to evaluate what is known and proven by the evidence concerning: (i) the incidence and (ii) early detection and follow-up strategies of neuropsychological sequelae in long-term cHL and DLBCL survivors.

## 2. Materials and Methods

This systematic review is part of a series of analyses exploring the management and follow-up of long-term lymphoma survivors to support FIL position statements. The scope of the position statements, as well as the clinical questions and population/intervention/control/outcome/study (PICOs) for each question, were discussed and agreed on by the FIL Long-Term Survivor Committee and presented at the FIL congress in 2019. We used the Preferred Reporting Items for Systematic Reviews and Meta-Analyses (PRISMA) guidelines to report the results [12].

### 2.1. Study Identification

MEDLINE (via PubMed), the Cochrane Library, and EMBASE were systematically searched for publications indexed between 1984 and November 2019, with no language or publication type restrictions. Search terms included extensive controlled vocabulary (MeSH and EMTREE) and free-text keywords, combining the conditions (Hodgkin disease, diffuse large B-cell lymphoma), interventions (e.g., chemotherapies and radiotherapy), and outcomes of interest (e.g., neuropathy, cognitive impairment, fatigue). Details on the search strategies can be found in the Supplementary Data (Table S1). We checked the reference lists of relevant studies to retrieve further studies and congress abstracts and searched study registries for unpublished or ongoing studies.

### 2.2. Eligibility Criteria

We included both primary studies (randomized controlled trials, prospective, and retrospective cohort studies and registry studies) and systematic reviews, including these study designs. We included studies involving long-term (≥5 years disease- or treatment-free), adult (≥18 years at diagnosis), cHL, or DLBCL survivors. In some cases, studies including not only patients with a follow-up ≥ 5 years or not only diagnosed in adult age were analyzed to extract data concerning a patient group fulfilling our inclusion criteria. Included studies assessed the incidence of neuropathy, chronic fatigue, cognitive impairment, and anxiety and depression. In parallel, we evaluated the existence and efficacy of follow-up strategies that prevent or manage the outcomes mentioned above.

Table 1 reports the clinical questions and corresponding PICOs addressed by this review.

The evaluation focused on patients treated with first-line therapy or second-line therapy, including autologous stem cell transplant (ASCT). Having received an allogeneic stem cell transplant was an exclusion criterion.

### 2.3. Risk of Bias and Quality of Evidence Assessment

We assessed the methodological quality of the included systematic reviews using the AMSTAR 2 tool [13], the risk of bias for the RCTs using the Cochrane risk-of-bias tool (RoB) [14] and the quality of cohort and registry studies using the Newcastle-Ottawa Scale [15]. The risk of bias and quality of evidence assessment was conducted by one reviewer and checked by another. Table S3 reports the risk of bias and quality of evidence assessment.

### 2.4. Study Selection and Data Extraction

One reviewer (SF or MAA) screened the title and abstract to select the studies, reviewed the full-text articles to confirm eligibility, and extracted the relevant information from the included trials. A second reviewer checked the eligibility and the data extraction to increase the accuracy of the process (CG). Any discrepancies were resolved by consensus and arbitration by a third author (CM).

Data collected from each study included the following 13 predefined items: (1) study identifier (first author, year of publication); (2) reference; (3) other publications; (4) study design; (5) population; (6) study duration; (7) follow-up; (8) sample size; (9) intervention/control group; (10) outcome measure; (11) main results; (12) conclusion; (13) risk of bias/quality assessment. A predefined spreadsheet (Excel 2007, Microsoft Corporation, Redmond, WA, USA^®^) was used for data extraction (Table S2).

### 2.5. Data Synthesis

As we expected a substantial degree of heterogeneity among the included studies, we did not pool data in the meta-analysis. For each clinical question, the included studies providing relevant information were summarized narratively and tabulated to highlight similarities and differences in their methods and results. We focused on the review outcomes listed in Table 1.

## 3. Results

### 3.1. Neurotoxicity: What Is the Incidence of PN in Long-Term cHL or DLBCL Survivors after First- and/or Second-Line CT or RT?

**Figure 1:**
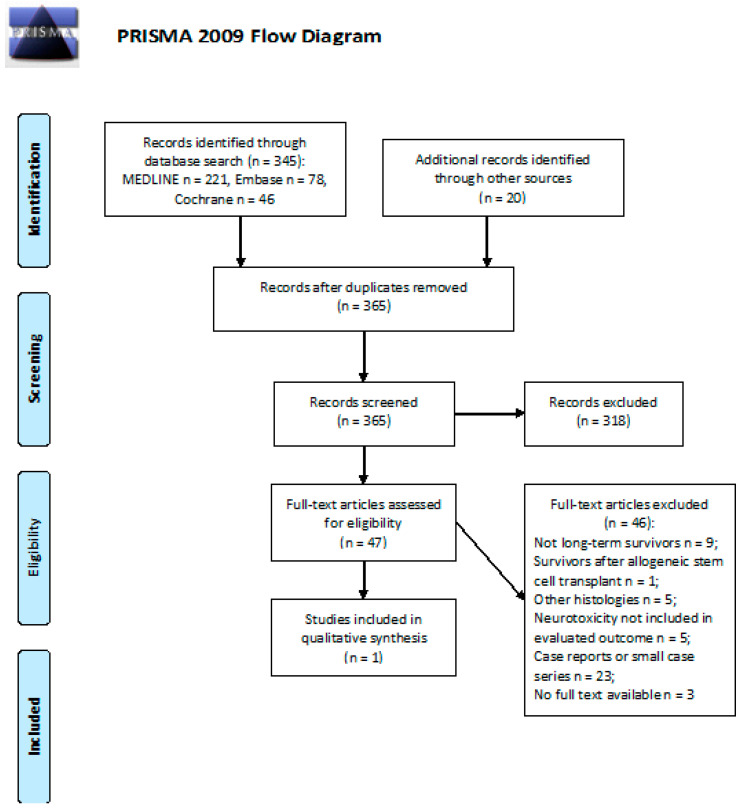
PRISMA flow diagram on peripheral neuropathy.

We screened 345 abstracts. A total of 20 additional publications were identified through other sources. There were 47 relevant publications retrieved as full texts. Of these, 46 were excluded. Details of the whole screening process, including reasons for full-text exclusion, are reported in Figure 1.

The only included study conducted by Oerlemans et al. [16] was a longitudinal survey among DLBCL patients registered in the Population-based HAematological Registry for Observational Studies (PHAROS), an extension of the Netherlands Cancer Registry. Patients were treated with rituximab (R-) cyclophosphamide-doxorubicin-vincristine-prednisone (CHOP) regimen, either every 14 or every 21 days or in a minority of cases with second-line chemotherapy followed by ASCT, between 2004 and 2010. The patients were asked to complete a questionnaire reporting health-related quality of life (HRQoL) and symptoms at the time of study inclusion and again one year later. A total of 256 DLBCL patients completed the first questionnaire. The mean time from diagnosis was 2.6 years (SD 1.3 years); 44% of patients had >24 months of follow-up after the end of treatment, and 93% underwent one treatment line. Of the 256 patients, 33% complained of tingling in the hands and feet, more often in those who were treated with R-CHOP14 than with R-CHOP21 (42% vs. 27%, *p* = 0.02). Worse scores on the EORTC Core Quality of Life questionnaire (EORTC QLQ-C30) were also reported. Data had been stratified for age since diagnosis, respectively 0–1 years after diagnosis (*n* = 66 patients), 1–2 years (*n* = 65 patients), 2–3 years (*n* = 53 patients), 3–5 years (*n* = 59 patients), and the incidence of this long term complication remained relatively stable over the time. The quality of this study (NOS) was high due to an optimal case definition and representativeness, good selection of controls, and outcome assessment. While this study did not report data only on long-term follow-up, it is the only one reporting PN in a homogeneous DLBCL patient cohort.

### 3.2. Neurotoxicity: Efficacy of Follow-Up Programs for Diagnosis and Management of PN in cHL or DLBCL Long-Term Survivors after First and/or Second Line CT or RT

**Figure 2:**
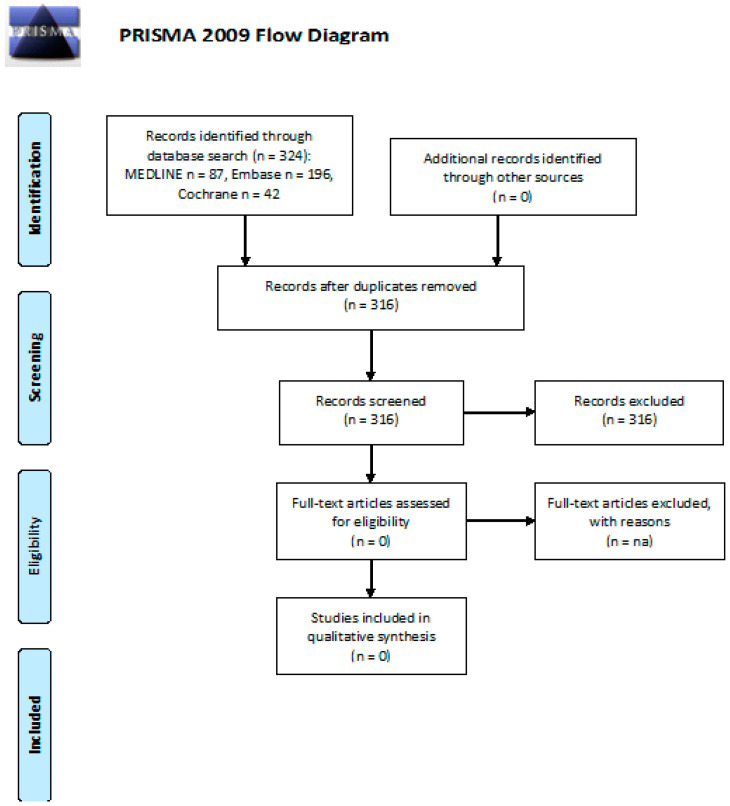
PRISMA flow diagram on the efficacy of follow-up programs for the diagnosis and management of peripheral neuropathy.

We screened 324 abstracts, with no relevant publications being retrieved as full texts. All publications were excluded from the final analysis. Details of the whole screening process are reported in Figure 2.

Based on current knowledge, we can assume that there is no evidence of validated follow-up strategies to detect, prevent, or improve symptoms of neuropathy in asymptomatic long-term cHL or DLBCL survivors.

### 3.3. Cognitive Impairment: What Is the Incidence of Cognitive Impairment in Long-Term cHL or DLBCL Survivors after First- and/or Second-Line CT or RT?

**Figure 3:**
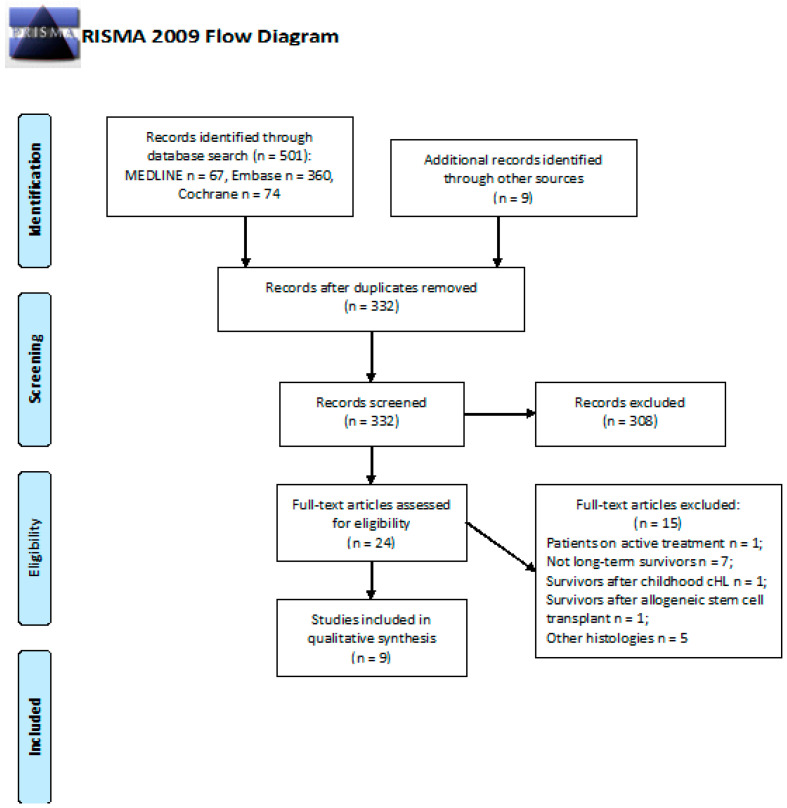
PRISMA flow diagram on the incidence of cognitive impairment.

We screened 501 abstracts, with 9 additional publications identified through other sources. There were 24 relevant publications retrieved as full texts; of these, 15 were excluded. Thus, 9 studies were included in the final sample and relative analysis. Details of the whole screening process, including reasons for full-text exclusion, are reported in Figure 3.

Of the 9 studies analyzed, there were no randomized controlled trials; there were 8 cross-sectional case-control studies and 1 prospective cohort study. The studies were conducted between 1995 and 2014. All the publications (*n* = 11) focused on cHL survivors.

In the study by Joly et al. [17], 93 long-term cHL survivors extracted from the Calvados General Tumor Registry (France) completed 2 mailed questionnaires (EORTC QLQ-C30 and a study questionnaire addressing education and social and family items). The mean time from diagnosis was 10 years (range 4–17). Collected data were compared to 186 matched healthy controls. The results showed impaired cognitive function in cHL survivors compared to healthy controls (79.5 vs. 89.8, *p* = 0.015), with the patients complaining of much or very much difficultly in concentrating on things (13% vs. 2%, *p* < 0.001) and very much difficulty in remembering things (20% vs. 6%, *p* <0.001). The quality of this study (NOS) was intermediate due to an optimal case definition and representativeness and a good selection of controls but a self-reported outcome assessment.

After this report, eight other studies [18,19,20,21,22,23,24,25] evaluated self-perceived cognitive function in a total of 1657 cHL survivors using the same instrument (EORTC QLQ-C30 questionnaire). Of these studies, one was a longitudinal cohort study, while the other seven were cross-sectional studies, with a control group present in four of them. Most of the patients included in the studies received first-line combined modality treatment; two studies evaluated self-perceived cognitive impairment incidence in long-term follow-up of relapsed/refractory cHL after ASCT [20,23]. The mean time from diagnosis ranged from 3.5 to 12 years. All but one study [19] reported lower cognitive function scores compared to healthy controls or the normative population. The quality of the studies was good in terms of selection and comparability criteria. Limitations concerned the absence of a control group in most of the studies, and the outcome evaluation was always carried out with written self-assessment tools.

No study evaluating cHL long-term survivors, both with a subjective evaluation through self-reported questionnaires, and an objective evaluation of the main cognitive domains (intelligence, attention, processing speed, memory and learning, executive function) performed by a neuropsychologist, could be found.

All the analyzed papers concerning cognitive impairment incidence after therapy in DLBCL were excluded because they both included heterogeneous histologies or median follow-up was shorter than 5 years, and/or data were not stratified for age since diagnosis. Therefore, based on current knowledge, we can assume that there are no clear data on the incidence of cognitive impairment in DLBCL long survivors.

### 3.4. Cognitive Impairment: Efficacy of Follow-Up Programs for Diagnosis and Management of Cognitive Impairment in cHL or DLBCL Long-Term Survivors after First and/or Second Line CT or RT

**Figure 4:**
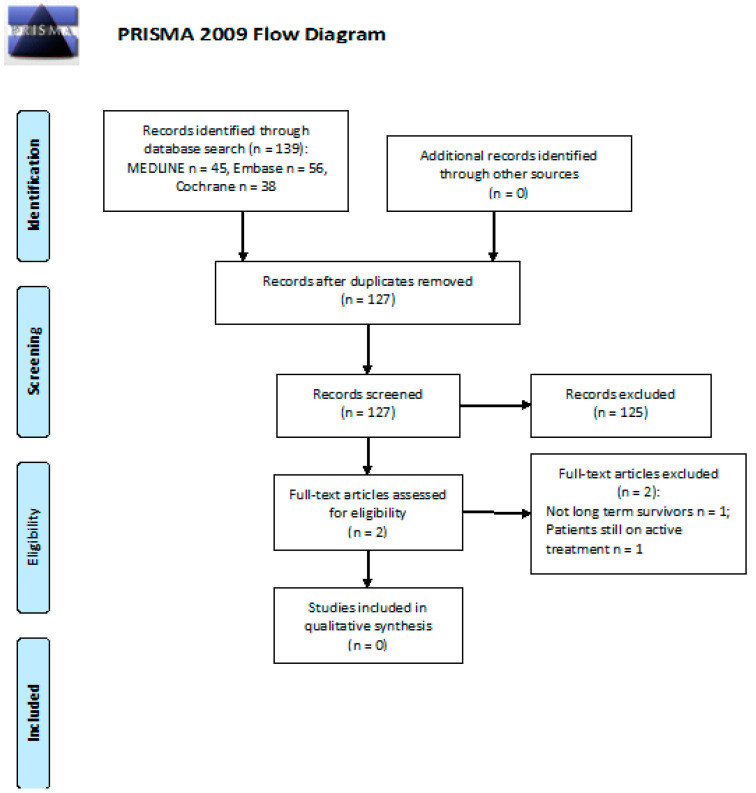
PRISMA flow diagram on the efficacy of follow-up programs for the diagnosis and management of cognitive impairment.

We screened 139 abstracts and retrieved 2 as full texts. All 139 publications were excluded from the final analysis. Details of the whole screening process, including reasons for full-text exclusion, are reported in Figure 4.

Based on this literature review, we can assume that, despite there being many questionnaires able to quantify self-perceived cognitive impairment, there were no validated follow-up strategies employing dedicated questionnaires or neuropsychological evaluation to diagnose, prevent or improve signs of cognitive impairment in asymptomatic long-term cHL or DLBCL survivors.

### 3.5. Fatigue: What Is the Incidence of Fatigue in Long-Term cHL or DLBCL Survivors after First- and/or Second-Line CT or RT?

**Figure 5:**
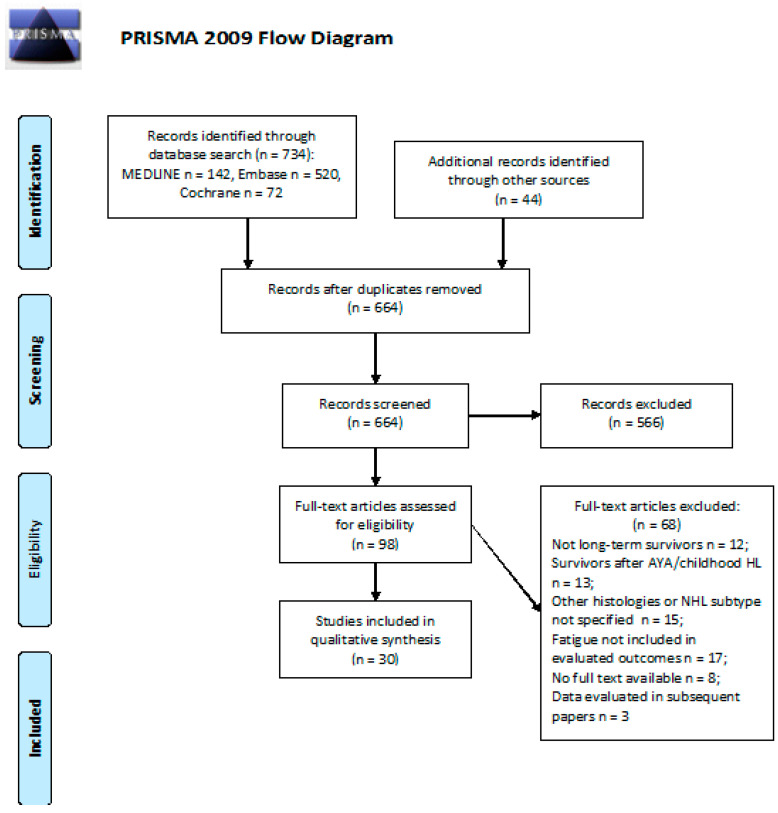
PRISMA flow diagram on the incidence of fatigue.

We screened 734 abstracts as well as an additional 44 publications identified through other sources. Of these 778 publications, we retrieved 98 as full texts. Of these, 68 were excluded; 30 studies were included in the final sample and relative analysis. Details of the whole screening process, including reasons for full-text exclusion, are reported in Figure 5.

Of the 32 included studies, 1 was a systematic review, 27 were cross-sectional studies, and 4 were longitudinal cohort studies conducted between 1993 and 2017. All publications focused on cHL survivors.

The systematic review by Linendoll et al. [6] focused on both the evaluation of persistent fatigue and HRQoL in cHL survivors. The authors analyzed 65 papers, of which only 27 [17,18,19,20,21,22,23,24,25,26,27,28,29,30,31,32,33,34,35,36,37,38,39,40,41,42,43] matched our search criteria. The 38 excluded papers mainly concerned either the late effects in pediatric populations (*n* = 13), focused on other HRQoL aspects (*n* = 18), or did not include cured patients or long-term survivors (*n* = 7). Of the included papers, two were longitudinal cohort studies, while the other 25 were cross-sectional studies, with a control group in 15 of them. The most commonly used multidimensional instrument for HRQoL evaluation was the EORTC QLQ-C30 questionnaire (*n* = 13). A total of 14 studies used fatigue-specific questionnaires (Fatigue Questionnaire, Multidimensional Fatigue Inventory—MFI, Fatigue Assessment Scale, FACIT-F). Results in this domain were homogeneous, with most of the studies indicating that cHL survivors were at increased risk of fatigue when compared to healthy controls. The mean fatigue prevalence among cHL survivors ranged from 10% to 43% [24,42]. Two studies evaluated fatigue incidence in long-term follow-up of relapsed/refractory cHL who underwent ASCT [20,23]: the mean time from ASCT was 10–12 years, and fatigue prevalence was the same as in other studies.

Among the studies included in the Linendoll review, worthy of note was a longitudinal cohort study assessing HRQoL in patients enrolled in the EORTC-GELA H8 trial in Europe [21]. The 935 included patients were assessed with the EORTC QLQ-C30, MFI, and a sexual function scale at the end of treatment and then serially for 10 years. The results indicated that the emotional function scores were more affected than were the physical function scores and that women reported lower HRQoL and increased symptom distress than did men. All the HRQoL domains they evaluated showed improvement within 18 months from treatment completion, with the exception of cognitive function and motivation. Further, high levels of fatigue at the end of treatment predicted persistent fatigue during long-term follow-up, with odds ratios ranging from 2.58 (95% CI 1.00–6.67) to 41.51 (95% CI 12.02–143.33; *p* ≤ 0.0001).

A global analysis of the results of Linendoll’s review revealed that several variables were correlated with higher fatigue scores: sociodemographics (older age, low education level), lymphoma features (presence of B symptoms at diagnosis), treatment type (combined modality treatment) and the presence of two or more comorbidities. In addition, a correlation between fatigue and late cardiopulmonary effects was noted, the association with thyroid dysfunction was controversial [29,34], and no association was evident with impaired gonadal function [29]. Two of the included studies also showed a positive correlation between chronic fatigue and depression, as evaluated by the Hospital Anxiety and Depression Scale (HADS) [24,29]. In the study by Daniels [24], 20% of cHL survivors reported both fatigue and anxiety, and 17% reported both fatigue and symptoms of depression. These scores were significantly lower in the norm population (8%, *p* < 0.001, and 9%, *p* = 0.004, respectively); the prevalence of fatigue among HL survivors with a high level of symptoms of depression was 97%, compared to 76% in the norm population [24]. Finally, one study [38] showed that an exercise intervention program had a positive impact on perceived fatigue (the total fatigue score decreased by 43.7%, from 21.5 to 12.1, after the intervention, *p* = 0.001).

The overall evaluation of the quality of this review was intermediate because, while the results were reported extensively, the explanation of the review methods and the relative inclusion/exclusion criteria was poor, and an adequate risk of bias evaluation of the included papers was lacking.

Our literature search yielded two worthy of note additional papers.

These were two longitudinal cohort studies evaluating a considerable number of cHL patients enrolled in the GHSG trials [44,45]. These two studies included the largest sample in our systematic review: respectively, 4981 and 4529 cHL patients who completed the QLQ-S questionnaire (EORTC QLQ-C30 and a question on current employment status) at the time of trial enrollment, then annually for up to 5 years. The analysis by Kreissl et al. [45] showed that the proportion of survivors with severe persistent fatigue (sFA = fatigue score ≥ 50) was 17% (95% CI 12–22) of patients with early-stage favorable disease, 27% (95% CI 22–32) of patients with early-stage unfavorable disease, and 22% (95% CI 12–32) of patients with advanced-stage disease. Behringer et al. [44] reported that the proportion of patients reporting sFA during survivorship remained relatively stable between year 1 (24%) and year 5 (20%) of follow-up. sFA was more frequent in female patients than in males at each time point analyzed. Other major findings emerging from the Behringer analysis were that sFA at baseline was correlated with PFS and treatment outcome, and that sFA during follow-up prevented survivors from social reintegration, thus having a negative correlation with employment status and financial problems.

The quality of the two last studies (NOS) was high due to an optimal case definition and representativeness, selection of controls and the use of validated, multidimensional assessment tools.

### 3.6. Anxiety and Depression: What Is the Incidence of Anxiety and Depression in Long-Term cHL or DLBCL Survivors after First- and/or Second-Line CT or RT?

**Figure 6:**
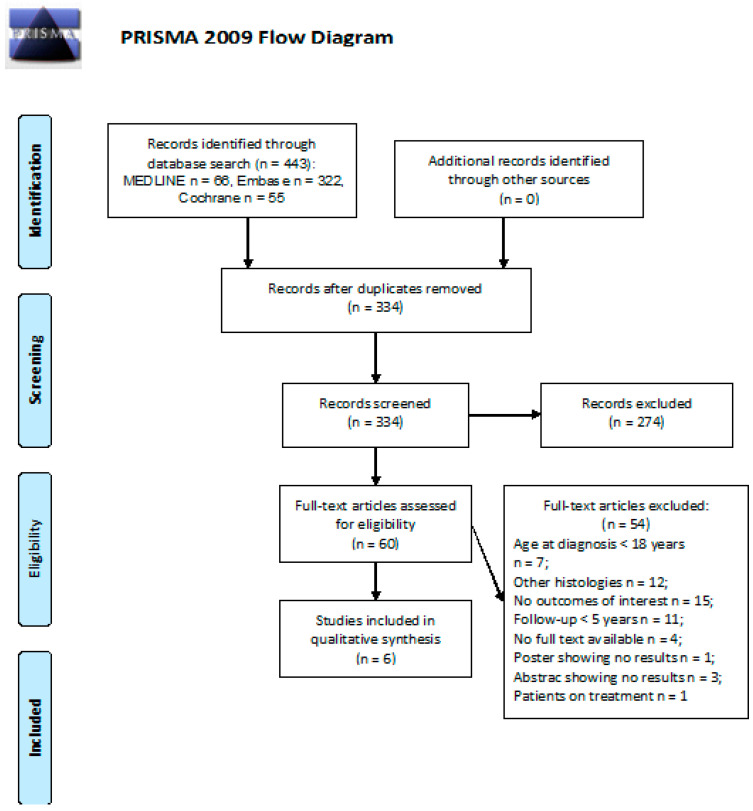
PRISMA flow diagram on the incidence of anxiety and depression.

We screened 443 abstracts; the full texts of 61 were retrieved. Of these, 55 were excluded, leaving 6 studies included in the final sample and relative analysis. Details of the whole screening process, including reasons for full-text exclusion, are reported in Figure 6.

All six included studies were cross-sectional studies [19,24,46,47,48,49], conducted between 1997 and 2017. All focused on cHL survivors.

A control group was present in three studies, and in all six, the incidence of anxiety and depression was assessed using the HADS questionnaire [50], a validated self-reported instrument. All six studies also tested other dimensions, such as fatigue and quality of life, and considered sociodemographic variables to assess correlations with psychological distress. All six studies included cHL survivors who had received different treatments (exclusive radiotherapy; exclusive chemotherapy; combined modality treatment).

Altogether, the results in this domain were not homogeneous, although the finding that cHL survivors are at increased risk for mood disturbance was highlighted.

In the already cited paper by Daniels et al. [24], 180 cHL survivors extracted from the Ehindoven Cancer Registry completed and returned the questionnaires. The mean time from diagnosis was 4.6 years, the mean age at the time of the survey was 46 years, and 55% received a combined modality treatment. A total of 23% of cHL survivors reported high levels of symptoms of anxiety and 18% of depression, compared with 13% and 12%, respectively, in the Dutch norm population. A correlation with fatigue was noted, in particular, a combination of fatigue and anxiety, occurring in 20% of cHL survivors and of fatigue and depression in 17%, compared with 8% and 9%, respectively, in the norm population (*p* < 0.001 and *p* = 0.004).

Loge et al. [31,47] conducted a national survey to assess the levels of psychological distress and to identify predictors of anxiety/depression after treatment. The survey, including the HADS questionnaire, was sent to 459 cHL survivors who had been treated with chemotherapy, radiotherapy, or both. The survey results reported an average anxiety score of 5.0 (SD = 4.1) and a mean depression score of 3.1 (SD = 3.3). Moreover, 27% of cHL survivors had anxiety and depression scores above the HADS cutoff value, with a higher percentage for the incidence of anxiety (14.5%) than for depression (4%) or for both dimensions (8.5%). Women had a higher incidence of anxiety than did men (28% vs. 20%, *p* = 0.054). Finally, anxiety and depression predictors were identified. Concerning anxiety, the time from diagnosis (observation period: 7 years or longer), type of treatment (combined modality treatment), low education level, and the presence of psychiatric symptoms before or during the illness were all predictors. Increasing age and the presence of psychiatric symptoms before cHL diagnosis predicted depression [47].

Magyari et al. [48] evaluated psychological distress and its risk factors in 163 cHL survivors. The patients included in the study underwent a standardized, validated, self-administered series of questionnaires, including HADS. Treatment data were acquired from hospital records. The authors detected a 25% rate of anxiety and a 10% rate of depression, similar to that found in the study by Loge et al. In this sample, the majority of cHL survivors were in early adulthood at the time of diagnosis, and their most important goal was to return to normal life and work after recovery. The survivors were divided into two subgroups based on employment status: Active (A) and Inactive (I). The results showed significantly higher anxiety scores in Subgroup I than in Subgroup A (I: M ± SD = 6.69 ± 3.64; A: M ± SD = 4.87 ± 3.35; *p* = 0.004). A significant difference was also reported for depression scores in the I and A subgroups (I: M ± SD = 4.87 ± 3.84; A: M ± SD = 2.17 ± 2.38; *p* < 0.001) [48].

Korszun et al. [46] aimed to assess the prevalence of depression and anxiety and fatigue in a sample of 780 hematological cancer survivors, including 280 cHL, 206 indolent NHL, 120 aggressive NHL, and 112 acute leukemia (AL). All the patients were treated at a single center in the UK over the course of 50 years. The results of the study showed a 15% rate of depression (cHL 14%; indolent NHL 17%; aggressive NHL 17%; AL 16%) at the time of questionnaire completion.

The previously cited study by Gil-Fernandez et al. [19] aimed to assess the quality of life and psychological well-being in a sample of 46 Spanish cHL patients. The authors did not detect any significant differences in cHL survivors in terms of the incidence of anxiety and depression (anxiety M ± SD = 6.17 ± 4.34, depression M ± SD = 3.41 ± 3.76) compared with that in the general population (anxiety M ± SD = 4.93 ± 3.58, depression M ± SD = 2.60 ± 2.97). As found in the study by Loge, higher anxiety scores in women were noted, and the rate of depression appeared to increase with age. However, these data had no statistical significance when compared with the general population (*p* = 0.16 and *p* = 0.07, respectively). No correlation was found with the stage of disease or with the type of treatment; a higher incidence of depression was found in the subgroup undergoing ASCT, although statistical significance was not reached (*p* = 0.08) [19].

In the sixth paper, by Aksnes et al. [49], the authors analyzed mental distress, fatigue, and QoL in survivors of bone tumors compared with gender- and age-matched samples of 362 cHL and testicular cancer survivors. The results showed that 25% of the 89 cHL survivors had anxiety and 8% depression. There were no statistically significant differences in the incidence of anxiety or depression among the three groups of cancer survivors.

The quality of the studies was good concerning selection (representativeness of the exposed cohort and demonstration that the outcome of interest was not present at the start of the study) and comparability (comparability of the cohorts based on the design or analysis). Limitations concerned the absence of a control group in three studies [46,47,48], and that the exposure evaluation was always carried out with written self-assessed tools.

### 3.7. Anxiety and Depression: Efficacy of Follow-Up Programs for Diagnosis and Management of Anxiety and Depression in cHL or DLBCL Long-Term Survivors after First and/or Second Line CT or RT

**Figure 7:**
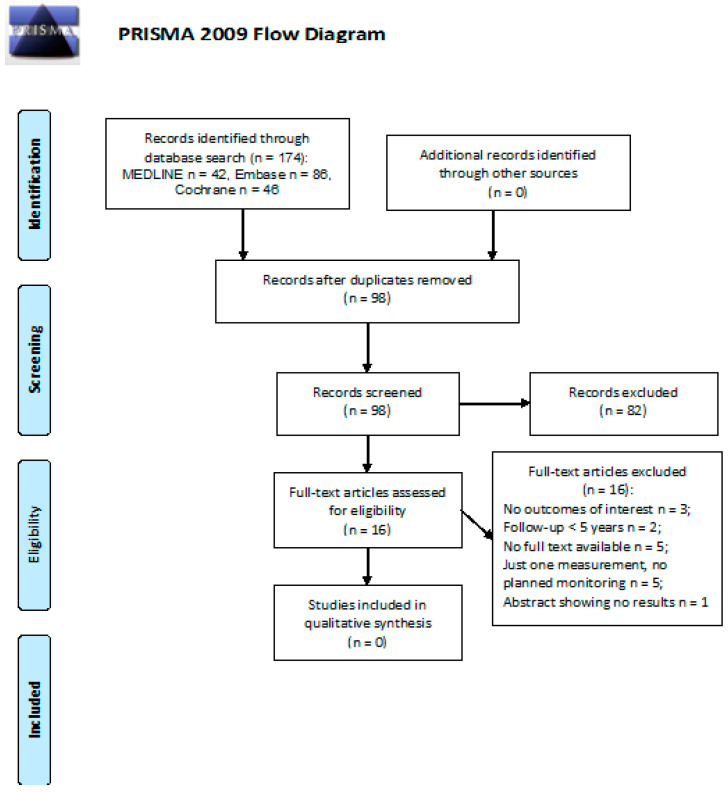
PRISMA flow diagram on the efficacy of follow-up programs for the diagnosis and management of anxiety and depression.

After screening 174 abstracts, we retrieved 16 relevant publications as full texts. These publications, however, were all excluded from the final analysis. Details of the whole screening process, including reasons for full-text exclusion, are reported in Figure 7.

Analyzing the articles selected during the screening process, we found that none of the records retrieved from the literature search had a planned follow-up scheme for anxiety and depression diagnosis and monitoring in the target population. This led us to exclude all of them.

## 4. Discussion

In recent years, the number of cancer survivors worldwide has significantly increased, and survivorship management has become a new challenge for modern oncology [51]. While survival rates have significantly improved in patients with cHL and DLBCL, some of these patients have to contend with long-term toxicities of anti-cancer treatment that can negatively impact the quality of life, comorbidity, and mortality [52].

It should also be emphasized that cHL (and less often DLBCL) is diagnosed at a young age and that this could have important consequences on psychophysical growth. For younger people with cHL, aggressive cancer treatment often interrupts important developmental milestones, such as graduation from high school, establishing relationships, finding a first apartment, or getting a job.

This systematic review aimed to assess the incidence and follow-up strategies of the neurological and psychological sequelae among long-term cHL and DLBCL survivors with respect to the PICOs detailed in Table 1.

Regarding peripheral neuropathy (PN), despite it being a well-described side effect of the most frequently used chemotherapy regimens in cHL and DLBCL treatment (ABVD, BV, or R-CHOP), we could not find any study objectively assessing its prevalence in long-term survivors. In our search for PN incidence, we examined several long-term cHL and DLBCL follow-up phase II studies and their safety analyses; most did not report data stratified by cured patients, did not include long-term survivors, or did not report long-term PN incidence. This was also true for publications concerning BV use in relapsed/refractory cHL [4,53,54]. Both the pivotal phase II study on long-term follow-up of BV and the safety analysis of phase III AETHERA study reported high sensory or motor PN incidence—grade 1–3 PN in up to 67% of treated patients, which almost completely resolved or improved within 6 months from the end of therapy. However, the reported data were not stratified for cured patients and could not, therefore, be analyzed for the aim of our systematic review. Concerning radiotherapy-induced neurological toxicity, two reviews [55,56] and one retrospective cohort study [57] collected case reports of late-onset PN, camptocormia, or “dropped head syndrome”. These three reports investigated cHL patients treated with large irradiation fields (extended, mantle, or total nodal irradiation) and higher radiotherapy doses. We excluded these studies from our analysis because of their methodology. A single study conducted by Frick et al., not included in our analysis because of methodological and bias limitations [58], showed a self-reported PN incidence of up to 33% of long-term cHL and NHL survivors. In this paper, data were not stratified by histology. Nevertheless, as 37% of the included patients were cHL survivors, and 89% of the entire study population was treatment-free, it can be assumed that these were “cured” patients. PN was assessed using an internet-based survivorship care plan (SCP) tool. It is well known that discrepancies exist between clinician- and patient-reported symptoms in oncology practice, with patients often reporting greater severity compared to what their clinician reports. However, the prevalence of PN in long-term survivors should not be underestimated, and clinicians should aim to design studies to assess it objectively.

Concerning the remaining neuropsychological sequelae (cognitive impairment, fatigue, anxiety and depression), our literature systematic review showed several limitations in the explored studies. First, the study population of almost all the studies analyzed was not homogeneous with respect to age at diagnosis and duration of survivorship, intended as time from diagnosis. The studies included both short- and long-term survivors, and the data were not stratified by different durations of survivorship. Moreover, several studies included patients diagnosed in pediatric age, in adolescence (the so-called adolescent and young adult—AYA), or in adulthood, and data were not stratified by age range. Second, several papers included different types of cancer, making it difficult to identify the specific long-term consequences of each neoplasm, which may differ according to the affected organ and the treatment undergone. Third, study methodology was often suboptimal; most of the analyzed papers were cross-sectional studies, and some lacked a control group. Although the NCCN Guidelines for Survivorship [11] recommend monitoring neuropsychological aspects over time, we found only four longitudinal trials assessing fatigue in cHL survivors [21,38,44,45], highlighting the difficulty in carrying out a good quality outcome assessment. Fourth, suboptimal study methodologies did not allow us to establish a causal relationship between the outcome of interest and the variables tested. Last, most of the analyzed studies focused on cHL survivors, while data on DLBCL survivors were scant. The incidence of neuropsychological sequelae in DLBCL, therefore, should have been extrapolated from the studies on NHL, most of which reported data not stratified by NHL subtype or which included patients with active disease. Because of those limitations, a great number of studies had to be excluded from our results analysis.

Despite the limitations encountered in the literature search, we can make some observations inferred by the included studies. Concerning cognitive impairment in cHL, nine studies showed a self-perceived lack of concentration and difficulty in remembering things. As observed for PN, discrepancies may exist between clinician- and patient-reported symptoms. We could find out only two studies, conducted by Trachtenberg et al. and Wouters et al. [59,60], assessing this outcome by means of an objective evaluation performed by a neuropsychologist. Both studies showed mild cognitive impairment in some cHL and aggressive NHL survivors, however, neither was included in the results analysis because of methodological limitations. It should be noted that in Wouters’ study [60], the subgroup of patients with deviant cognitive performance did not complain substantially more often of poor cognition than the other patients did. This analysis underlines the importance of assessing cognitive function using validated neuropsychological tests in addition to self-reports [60]. However, these studies did not include only long-term cHL or DLBCL survivors, thus we cannot draw any definite conclusions in this setting.

Fatigue was the most extensively studied outcome of interest among the neuropsychological sequelae in cHL survivors. The mean prevalence of fatigue among cHL survivors ranged from 10% to 43% [24,42]. Worthy of note among the included papers is a longitudinal cohort study evaluating a large number of cHL patients enrolled in the GHSG trials [44]. This study showed that the proportion of patients reporting sFA during survivorship remained relatively stable from year 1 (24% of survivors) to year 5 (20% of survivors) of follow-up. Moreover, two major findings emerged from the analysis and should be emphasized: sFA at baseline is correlated with PFS and treatment outcome, and sFA during follow-up prevents survivors from social reintegration, as it has a negative correlation with employment status and financial problems [44]. This report, given the young age of cHL patients at diagnosis and the potential social impact of sFA, should induce clinicians to recognize and manage this symptom promptly.

Concerning the last outcome, the incidence of anxiety and depression, evaluated with HADS in cHL long-term survivors did not appear homogeneous in the analyzed studies.

Although several monitoring tools are available for these late sequelae, our literature search brought to light no data on monitoring and follow-up strategies of PN and other neuropsychological sequelae that have been validated by solid evidence. To that end, numerous SCPs have been developed and used as a means to promote coordinated survivorship care. Many are publicly available internet-based SCP tools. None, however, is a validated tool that the hematology community has reached a consensus on and uses regularly.

Despite continuous improvement in the cure rates of cHL and DLBCL, we clearly still need to learn how to successfully manage HRQoL during diagnosis, treatment, and follow-up. Assessing the long-term effects of cancer and its treatment is essential to patient care. Several papers analyzed in our systematic review reflect the great sensitivity toward this aspect of cancer and lymphoma care in northern Europe, where many trials assessing the long-term outcomes in lymphoma survivors have been conducted. In particular, three [16,26,43] have been conceived within the scope of the Population-based HAematological Registry for Observational Studies (PHAROS), an extension of the Netherlands Cancer Registry (NCR), and PROFILES (Patient Reported Outcomes Following Initial treatment and Long term Evaluation of Survivorship) program within the Eindhoven Cancer Registry (ECR). Although overall survival and disease-free survival remain key endpoints for randomized clinical trials in oncology, patient-reported outcomes (PROs) are increasingly used to inform treatment decisions and enhance the quality of care. As a result, PRO assessments are more frequently being incorporated into randomized controlled trials in oncology. PROs describe patients’ physical, emotional, functional, and psychosocial well-being and can provide help in assessing the cumulative impact that cancer and its treatment have on patients. However, few studies conducted with this methodology are now currently available, and none of them include cHL or DLBCL long-term survivors.

## 5. Conclusions

In conclusion, the assessment of PN and other neuropsychological sequelae in long-term cHL and DLBCL survivors remains to be adequately dealt with. There is a dearth of information regarding the incidence of these long-term sequelae, in particular, in DLBCL patients, and better studies (both in objectives and methodology) should be conducted with the specific focus of determining them.

Based on our observations, future trials in this setting should aim to: (1) design studies including well-defined populations in terms of age at diagnosis, duration of survivorship, and lymphoma subtype, stratifying results by age, duration of survivorship, and disease activity; (2) promote longitudinal trial design to assess the outcomes of interest over time and to develop follow-up programs that can be translated into daily practice. In particular, in studies including DLBCL patients that more often need second-line therapies than cHL, observation time since diagnosis should be prolonged to fit the definition of long-term survivors. For this purpose, it may be feasible to extend the outcomes and prolong long-term toxicity assessment (including PN or anxiety and depression incidence) in selected phase II trials, which generally include well-defined study populations.

Despite the lack of data, the FIL researchers agree that an early interception of all symptoms in the neuropsychological area and their regular assessment is crucial to the recovery and long-term management of life after cancer. To this aim, the best method for early detection and monitoring has yet to be defined by future research for the specific subset of cHL and DLBCL long-term survivors.

## Figures and Tables

**Table 1:** Clinical questions and PICOs addressed by the review: neurological and mood disorders.

Clinical Question	PICOs
What is the incidence of PN in long-term cHL or DLBCL survivors after first- and/or second-line CT or RT?	P: long-term cHL or DLBCL survivor (≥5 years disease- or treatment-free) adults (≥18-year-old at diagnosis) treated with first-line therapy or second-line therapy including ASCT I: chemotherapy (e.g., ABVD for cHL or R-CHOP for DLBCL), radiotherapy C: none/age- and sex-matched general population/other chemotherapy or radiotherapy regimen O: incidence of sensory/motor neuropathy cases, other neurotoxicities S: cohort, controlled cohort, RCT, reviews of those studies
Efficacy of follow-up programs for diagnosis and management of PN in cHL or DLBCL long-term survivors after first and/or second line CT or RT	P: long-term cHL or DLBCL survivor (≥5 years disease- or treatment-free) adults (≥18 years at diagnosis) treated with first-line therapy or second-line therapy including ASCT I: chemotherapy (e.g., ABVD for cHL or R-CHOP for DLBCL), radiotherapy C: scheduled follow-up program/no scheduled follow-up program/scheduled follow-up program with different timing (frequency or other variables) O: incidence of sensory/motor neuropathy cases, other neurotoxicities, QoL S: cohort, controlled cohort, RCT, reviews of those studies
What is the incidence of cognitive impairment in long-term cHL or DLBCL survivors after first- and/or second-line CT or RT?	P: long-term cHL or DLBCL survivor (≥5 years disease- or treatment-free) adults (≥18 years at diagnosis) treated with first-line therapy or second-line therapy including ASCT I: chemotherapy (e.g., ABVD for cHL or R-CHOP for DLBCL), radiotherapy C: none/age- and sex-matched general population/other chemotherapy or radiotherapy regimen O: incidence of cognitive impairment (e.g., lack of memory, concentration, attention) S: cohort, controlled cohort, RCT, reviews of those studies
Efficacy of follow-up programs for diagnosis and management of cognitive impairment in cHL or DLBCL long-term survivors after first and/or second line CT or RT	P: long-term cHL or DLBCL survivor (≥5 years disease- or treatment-free) adults (≥18 years at diagnosis) treated with first-line therapy or second-line therapy including ASCT I: chemotherapy (e.g., ABVD for cHL or R-CHOP for DLBCL), radiotherapy C: scheduled follow-up program/no scheduled follow-up program/scheduled follow-up program with different timing (frequency or other variables) O: incidence of cognitive impairment, QoL S: cohort, controlled cohort, RCT, reviews of those studies
What is the incidence of fatigue in long-term cHL or DLBCL survivors after first and/or second line CT or RT?	P: long-term cHL or DLBCL survivor (≥5 years disease- or treatment-free) adults (≥18 years at diagnosis) treated with first-line therapy or second-line therapy including ASCT I: chemotherapy (e.g., ABVD for cHL or R-CHOP for DLBCL), radiotherapy C: none/age- and sex-matched general population/other chemotherapy or radiotherapy regimen O: incidence/prevalence of fatigue S: cohort, controlled cohort, RCT, reviews of those studies
What is the incidence of anxiety and depression in long-term cHL or DLBCL survivors after first- and/or second-line CT or RT?	P: long-term cHL or DLBCL survivor (≥5 years disease- or treatment-free) adults (≥18 years at diagnosis) treated with first-line therapy or second-line therapy including ASCT I: chemotherapy (e.g., ABVD for cHL or R-CHOP for DLBCL), radiotherapy C: none/age- and sex-matched general population/other chemotherapy or radiotherapy regimen O: incidence of anxiety and depression S: cohort, controlled cohort, RCT, reviews of those studies
Efficacy of follow-up programs for diagnosis and management of anxiety and depression in cHL or DLBCL long-term survivors after first and/or second line CT or RT	P: long-term cHL or DLBCL survivor (≥5 years disease or treatment-free) adults (≥18 years at diagnosis) treated with first-line therapy or second-line therapy including ASCT I: chemotherapy (e.g., ABVD for cHL or R-CHOP for DLBCL), radiotherapy C: scheduled follow-up program/no scheduled follow-up program/scheduled follow-up program with different timing (frequency or other variables) O: incidence of anxiety and depression, QoL S: cohort, controlled cohort, RCT, reviews of those studies

cHL, classical Hodgkin lymphoma; DLBCL, diffuse large B-cell lymphoma; ASCT, autologous stem cell transplant; ABVD, doxorubicin-bleomycin-vinblastine-dacarbazine; R-CHOP, rituximab-cyclophosphamide-doxorubicin-vincristine-prednisone; QoL, quality of life, P, population; I, intervention; C, control; O, outcome.

## Data Availability

Not applicable.

## Supplementary Materials

The following are available online at https://www.mdpi.com/article/10.3390/cancers13143401/s1, Table S1: Search strategies, Table S2: Full-text eligibility and data extraction, Table S3: Risk of bias.
