# Supplementary material for: Late Neurological and Cognitive Sequelae and Long-Term Monitoring of Classical Hodgkin Lymphoma and Diffuse Large B-Cell Lymphoma Survivors: A Systematic Review by the Fondazione Italiana Linfomi
Source: Cancers (Basel). 2021 Jul 7;13(14):3401. doi: 10.3390/cancers13143401 (PMC8307605; doi:10.3390/cancers13143401)
Supplement: Supplementary file 1 [file cancers-13-03401-s001.zip › Table S1_Search strategies.pdf]

Supplementary mat.1: **Example of search strategy used in MedLine and adapted to search the other databases.**

- PICO Neuropathy incidence

MedLine, Embase, and Cochrane databases up to November 2019

("hodgkin disease" OR hodgkin disease[mh] OR Lymphoma, Large B-Cell, Diffuse[MH] OR DLBCL[TIAB] OR "diffuse large B cell lymphoma" OR "HODGKIN LYMPHOMA") AND (Survivors[mh] OR SURVIV*[tiab] OR Disease-Free Survival[mh]) AND (Antineoplastic Agents[MH] OR "Antineoplastic Protocols"[Mesh] OR "Chemoradiotherapy"[Mesh] OR "Chemotherapy, Adjuvant"[Mesh] OR "Consolidation Chemotherapy"[Mesh] OR "Antineoplastic Combined Chemotherapy Protocols"[Mesh] OR "Induction Chemotherapy"[Mesh] OR CHEMOTHERAP*[TIAB] OR TREATMENT[TIAB] OR THERAPY OR ABVD[TIAB] OR DOXORUBICIN OR BLEOMYCIN OR VINBLASTINE OR DACARBAZINE OR "RCHOP" OR CHOP OR rituximab OR cyclophosphamide OR doxorubicin OR vincristine OR prednisone OR radiotherapy OR "high dose chemotherapy" OR "autologous stem cell graft" OR "autologous stem cell grafting" OR "autologous stem cell transplant" OR "autologous stem cell transplantation" OR "hematopoietic stem cell tranplantation" OR "hematopoietic stem cell transplant" OR transplant OR transplantation OR BRENTUXIMAB OR Stem Cell Transplantation[MH] AND ("Neurologic Manifestations"[Mesh] OR "Peripheral Nervous System Diseases"[Mesh] OR "Neurotoxicity Syndromes"[Mesh] OR neuropath* OR neurotoxic* OR "neurological impairment" OR "neurological impairment" OR "peripheral nervous damage" OR "peripheral nervous diseases" OR "peripheral nervous disorder" OR "peripheral nervous disorders" OR "peripheral nervous disturbances" OR "peripheral nervous dysfunction" OR "peripheral nervous effects" OR "peripheral nervous manifestations" OR "peripheral nervous symptoms" OR "peripheral nervous system" OR "peripheral nervous system damage" OR "peripheral nervous system degeneration" OR "peripheral nervous system disorder" OR "peripheral nervous system disorders" OR "peripheral nervous system disturbances" OR "peripheral nervous system dysfunction" OR "peripheral nervous system impairment" OR "peripheral nervous system impairments")

- PICO Cognitive decline incidence

MedLine, Embase, and Cochrane databases up to October 2019

("hodgkin disease" OR hodgkin disease[mh] OR Lymphoma, Large B-Cell, Diffuse[MH] OR DLBCL[TIAB] OR "diffuse large B cell lymphoma" OR "HODGKIN LYMPHOMA") AND (Survivors[mh] OR SURVIV*[tiab] OR Disease-Free Survival[mh]) AND (Cognition Disorders[MH] OR Neurobehavioral Manifestations[mh] OR Attention[MH] OR Cognition[mh] OR Neuropsychological Tests[mh] OR Executive Function[MH] OR "executive functions"[tiab] OR ((cognit* OR neurocognit* OR neuropsycholog* OR memory OR neurobehavior* OR neurobehaviour*) AND (impair* OR deficit* OR declin* OR disorder* OR function* OR dysfunction* OR decrement* OR disturb* OR problem* OR sequelae* OR assess*))) AND (Antineoplastic Agents[MH] OR "Antineoplastic Protocols"[Mesh] OR "Chemoradiotherapy"[Mesh] OR "Chemotherapy, Adjuvant"[Mesh] OR "Consolidation Chemotherapy"[Mesh] OR "Antineoplastic Combined Chemotherapy Protocols"[Mesh] OR "Induction Chemotherapy"[Mesh] OR CHEMOTHERAP*[TIAB] OR TREATMENT[TIAB] OR THERAPY OR ABVD[TIAB] OR DOXORUBICIN OR BLEOMYCIN OR VINBLASTINE OR DACARBAZINE OR "RCHOP" OR CHOP OR rituximab OR cyclophosphamide OR doxorubicin OR vincristine OR prednisone OR radiotherapy)

- PICO fatigue incidence

MedLine, Embase, and Cochrane databases up to October 2020

("hodgkin disease" OR hodgkin disease[mh] OR Lymphoma, Large B-Cell, Diffuse[MH] OR DLBCL[TIAB] OR "diffuse large B cell lymphoma" OR "HODGKIN LYMPHOMA") AND (Survivors[mh] OR SURVIV*[tiab] OR Disease-Free Survival[mh]) AND (fatigue[mh] OR fatigue) AND ("Induction Chemotherapy"[Mesh] OR "high dose chemotherapy" OR "autologous stem cell graft" OR "autologous stem cell grafting" OR "autologous stem cell transplant" OR "autologous stem cell transplantation" OR "hematopoietic stem cell tranplantation" OR "hematopoietic stem cell transplant" OR transplant OR transplantation OR BRENTUXIMAB OR Stem Cell Transplantation[MH] OR Antineoplastic Agents[MH] OR "Antineoplastic Protocols"[Mesh] OR "Chemoradiotherapy"[Mesh] OR "Chemotherapy, Adjuvant"[Mesh] OR "Consolidation Chemotherapy"[Mesh] OR "Antineoplastic Combined Chemotherapy Protocols"[Mesh] OR "Induction Chemotherapy"[Mesh] OR CHEMOTHERAP*[TIAB] OR TREATMENT[TIAB] OR THERAPY OR ABVD[TIAB] OR DOXORUBICIN OR BLEOMYCIN OR VINBLASTINE OR DACARBAZINE OR "RCHOP" OR CHOP OR rituximab OR cyclophosphamide OR doxorubicin OR vincristine OR prednisone OR radiotherapy)

- PICO anxiety and depression incidence

MedLine, Embase, and Cochrane databases up to October 2019

("hodgkin disease" OR hodgkin disease[mh] OR Lymphoma, Large B-Cell, Diffuse[MH] OR DLBCL[TIAB] OR "diffuse large B cell lymphoma" OR "HODGKIN LYMPHOMA") AND (Survivors[mh] OR SURVIV*[tiab] OR Disease-Free Survival[mh]) AND ("Anxiety"[Mesh] OR "Anxiety Disorders"[Mesh] OR anxi*[tiab] OR "Depression"[Mesh] OR depression[tiab] OR depressive[tiab] OR "Mood Disorders"[Mesh]) AND ("Induction Chemotherapy"[Mesh] OR "high dose chemotherapy" OR "autologous stem cell graft" OR "autologous stem cell grafting" OR "autologous stem cell transplant" OR "autologous stem cell transplantation" OR "hematopoietic stem cell tranplantation" OR "hematopoietic stem cell transplant" OR transplant OR transplantation OR BRENTUXIMAB OR Stem Cell Transplantation[MH] OR Antineoplastic Agents[MH] OR "Antineoplastic Protocols"[Mesh] OR "Chemoradiotherapy"[Mesh] OR "Chemotherapy, Adjuvant"[Mesh] OR "Consolidation Chemotherapy"[Mesh] OR "Antineoplastic Combined Chemotherapy Protocols"[Mesh] OR "Induction Chemotherapy"[Mesh] OR CHEMOTHERAP*[TIAB] OR TREATMENT[TIAB] OR THERAPY OR ABVD[TIAB] OR DOXORUBICIN OR BLEOMYCIN OR VINBLASTINE OR DACARBAZINE OR "RCHOP" OR CHOP OR rituximab OR cyclophosphamide OR doxorubicin OR vincristine OR prednisone OR radiotherapy)

- PICO neuropathy follow-up

MedLine, Embase, and Cochrane databases up to October 2019

("hodgkin disease" OR hodgkin disease[mh] OR Lymphoma, Large B-Cell, Diffuse[MH] OR DLBCL[TIAB] OR "diffuse large B cell lymphoma" OR "HODGKIN LYMPHOMA") AND (Survivors[mh] OR follow up studies[mh] OR SURVIVOR*[tiab] OR Long-Term Care[mh] OR Disease-Free Survival[mh]) AND ("Neurologic Manifestations"[Mesh] OR "Peripheral Nervous System Diseases"[Mesh] OR "Neurotoxicity Syndromes"[Mesh] OR neuropath* OR neurotoxic* OR "neurological impairment" OR "neurological impairment" OR "peripheral nervous damage" OR "peripheral nervous diseases" OR "peripheral nervous disorder" OR "peripheral nervous disorders" OR "peripheral nervous disturbances" OR "peripheral nervous dysfunction" OR "peripheral nervous effects" OR "peripheral nervous manifestations" OR "peripheral nervous symptoms" OR "peripheral nervous system" OR "peripheral nervous system damage" OR "peripheral nervous system degeneration" OR "peripheral nervous system disorder" OR "peripheral nervous system disorders" OR "peripheral nervous system disturbances" OR "peripheral nervous system dysfunction" OR "peripheral nervous system impairment" OR "peripheral nervous system impairments") AND ("Induction Chemotherapy"[Mesh] OR "high dose chemotherapy" OR "autologous stem cell graft" OR "autologous stem cell grafting" OR "autologous stem cell transplant" OR "autologous stem cell transplantation" OR "hematopoietic stem cell tranplantation" OR "hematopoietic stem cell transplant" OR transplant OR transplantation OR BRENTUXIMAB OR Stem Cell Transplantation[MH] OR Antineoplastic Agents[MH] OR "Antineoplastic Protocols"[Mesh] OR "Chemoradiotherapy"[Mesh] OR "Chemotherapy, Adjuvant"[Mesh] OR "Consolidation Chemotherapy"[Mesh] OR "Antineoplastic Combined Chemotherapy Protocols"[Mesh] OR "Induction Chemotherapy"[Mesh] OR CHEMOTHERAP*[TIAB] OR TREATMENT[TIAB] OR THERAPY OR ABVD[TIAB] OR DOXORUBICIN OR BLEOMYCIN OR VINBLASTINE OR DACARBAZINE OR "RCHOP" OR CHOP OR rituximab OR cyclophosphamide OR doxorubicin OR vincristine OR prednisone OR radiotherapy) AND ("Aftercare"[Mesh] aftercare [tiab] OR "Secondary Care"[Mesh] OR "Secondary Care" OR "Continuity of Patient Care"[Mesh] OR "Continuity of Patient Care" OR "Patient Care Management"[Mesh] OR "Patient Care Management" OR "patient care planning" OR "Health Planning Guidelines"[Mesh] OR "follow up" OR "late side effect" OR "late side effects" OR "late adverse effect" OR "late adverse effects" OR "late adverse event" OR "late adverse event rates" OR "late adverse events" OR "late adverse health effects" OR "late adverse reaction" OR "late adverse reactions" OR "late adverse side effects" OR "late adverse toxicity" OR "late onset" OR "late diagnosis" OR "long term" OR "patient care plan" OR "patient care planning" OR "patient surveillance" OR surveillance[tiab] OR "patient monitoring" OR ((diagnosis[mh] OR diagnosis[sh] OR diagnos*[tiab]) AND (long OR late)))

- PICO cognitive decline follow up

MedLine, Embase, and Cochrane databases up to October 2019

("hodgkin disease" OR hodgkin disease[mh] OR Lymphoma, Large B-Cell, Diffuse[MH] OR DLBCL[TIAB] OR "diffuse large B cell lymphoma" OR "HODGKIN LYMPHOMA") AND (Survivors[mh] OR SURVIV*[tiab] OR Disease-Free Survival[mh]) AND (Cognition Disorders [MH] OR Neurobehavioral Manifestations[mh] OR Attention[MH] OR Cognition[mh] OR Neuropsychological Tests[mh] OR Executive Function[MH] OR "executive functions"[tiab] OR ((cognit* OR neurocognit* OR neuropsycholog* OR memory OR neurobehavior* OR neurobehaviour*) AND (test OR impair* OR deficit* OR declin* OR disorder* OR function* OR dysfunction* OR decrement* OR disturb* OR problem* OR sequelae* OR assess*))) AND ("Induction Chemotherapy"[Mesh] OR "high dose chemotherapy" OR "autologous stem cell graft" OR "autologous stem cell grafting" OR "autologous stem cell transplant" OR "autologous stem cell transplantation" OR "hematopoietic stem cell tranplantation" OR "hematopoietic stem cell transplant" OR transplant OR transplantation OR BRENTUXIMAB OR Stem Cell Transplantation[MH] OR Antineoplastic Agents[MH] OR "Antineoplastic Protocols"[Mesh] OR "Chemoradiotherapy"[Mesh] OR "Chemotherapy, Adjuvant"[Mesh] OR "Consolidation Chemotherapy"[Mesh] OR "Antineoplastic Combined Chemotherapy Protocols"[Mesh] OR "Induction Chemotherapy"[Mesh] OR CHEMOTHERAP*[TIAB] OR TREATMENT[TIAB] OR THERAPY OR ABVD[TIAB] OR DOXORUBICIN OR BLEOMYCIN OR VINBLASTINE OR DACARBAZINE OR "RCHOP" OR CHOP OR rituximab OR cyclophosphamide OR doxorubicin OR vincristine OR prednisone OR radiotherapy) AND (follow up studies[mh] OR "follow up" OR Long-Term Care[mh] OR "Aftercare"[Mesh] aftercare [tiab] OR "Secondary Care"[Mesh] OR "Secondary Care" OR "Continuity of Patient Care"[Mesh] OR "Continuity of Patient Care" OR "Patient Care Management"[Mesh] OR "Patient Care Management" OR "Patient Care Planning"[Mesh] OR "patient care planning" OR "Health Planning Guidelines"[Mesh] OR "follow up" OR "late side effect" OR "late side effects" OR "late adverse effect" OR "late adverse effects" OR "late adverse event" OR "late adverse event rates" OR "late adverse events" OR "late adverse health effects" OR "late adverse reaction" OR "late adverse reactions" OR "late adverse side effects" OR "late adverse toxicity" OR "late onset" OR "late diagnosis" OR "long term" OR "patient care plan" OR "patient care planning" OR "patient surveillance" OR surveillance[tiab] OR "patient monitoring" OR ((diagnosis[mh] OR diagnosis[sh] OR diagnos*[tiab]) AND (long OR late)))

- PICO anxiety and depression follow up

MedLine, Embase, and Cochrane databases up to October 2019

("hodgkin disease" OR hodgkin disease[mh] OR Lymphoma, Large B-Cell, Diffuse[MH] OR DLBCL[TIAB] OR "diffuse large B cell lymphoma" OR "HODGKIN LYMPHOMA") AND (Survivors[mh] OR SURVIV*[tiab] OR Disease-Free Survival[mh]) AND ("Anxiety"[Mesh] OR "Anxiety Disorders"[Mesh] OR anxi*[tiab] OR "Depression"[Mesh] OR depression[tiab] OR depressive[tiab] OR "Mood Disorders"[Mesh]) AND ("Induction Chemotherapy"[Mesh] OR "high dose chemotherapy" OR "autologous stem cell graft" OR "autologous stem cell grafting" OR "autologous stem cell transplant" OR "autologous stem cell transplantation" OR "hematopoietic stem cell tranplantation" OR "hematopoietic stem cell transplant" OR transplant OR transplantation OR BRENTUXIMAB OR Stem Cell Transplantation[MH] OR Antineoplastic Agents[MH] OR "Antineoplastic Protocols"[Mesh] OR "Chemoradiotherapy"[Mesh] OR "Chemotherapy, Adjuvant"[Mesh] OR "Consolidation Chemotherapy"[Mesh] OR "Antineoplastic Combined Chemotherapy Protocols"[Mesh] OR "Induction Chemotherapy"[Mesh] OR CHEMOTHERAP*[TIAB] OR TREATMENT[TIAB] OR THERAPY OR ABVD[TIAB] OR DOXORUBICIN OR BLEOMYCIN OR VINBLASTINE OR DACARBAZINE OR "RCHOP" OR CHOP OR rituximab OR cyclophosphamide OR doxorubicin OR vincristine OR prednisone OR radiotherapy) AND (follow up studies[mh] OR "follow up" OR Long-Term Care[mh] OR "Aftercare"[Mesh] aftercare [tiab] OR "Secondary Care"[Mesh] OR "Secondary Care" OR "Continuity of Patient Care"[Mesh] OR "Continuity of Patient Care" OR "Patient Care Management"[Mesh] OR "Patient Care Management" OR "Patient Care Planning"[Mesh] OR "patient care planning" OR "Health Planning Guidelines"[Mesh] OR "follow up" OR "late side effect" OR "late side effects" OR "late adverse effect" OR "late adverse effects" OR "late adverse event" OR "late adverse event rates" OR "late adverse events" OR "late adverse health effects" OR "late adverse reaction" OR "late adverse reactions" OR "late adverse side effects" OR "late adverse toxicity" OR "late onset" OR "late diagnosis" OR "long term" OR "patient care plan" OR "patient care planning" OR "patient surveillance" OR surveillance[tiab] OR "patient monitoring" OR ((diagnosis[mh] OR diagnosis[sh] OR diagnos*[tiab]) AND (long OR late)))
